# Genomic biomarker of checkpoint efficacy, highways for precision medicine in lung cancer

**DOI:** 10.18632/oncotarget.26389

**Published:** 2018-11-27

**Authors:** Francois Ghiringhelli, Caroline Truntzer

**Affiliations:** Francois Ghiringhelli: Platform of Transfer in Cancer Biology, Georges François Leclerc Cancer Center, UNICANCER, Dijon, France; Univ. Bourgogne Franche-Comté, Dijon, France; Georges François Leclerc Cancer Center - UNICANCER, Dijon, France; INSERM U1231, Dijon, France; Genetic and Immunology Medical Institute, Dijon, France

**Keywords:** lung cancer, immune checkpoint, exome, biomarker

**Figure 1:**
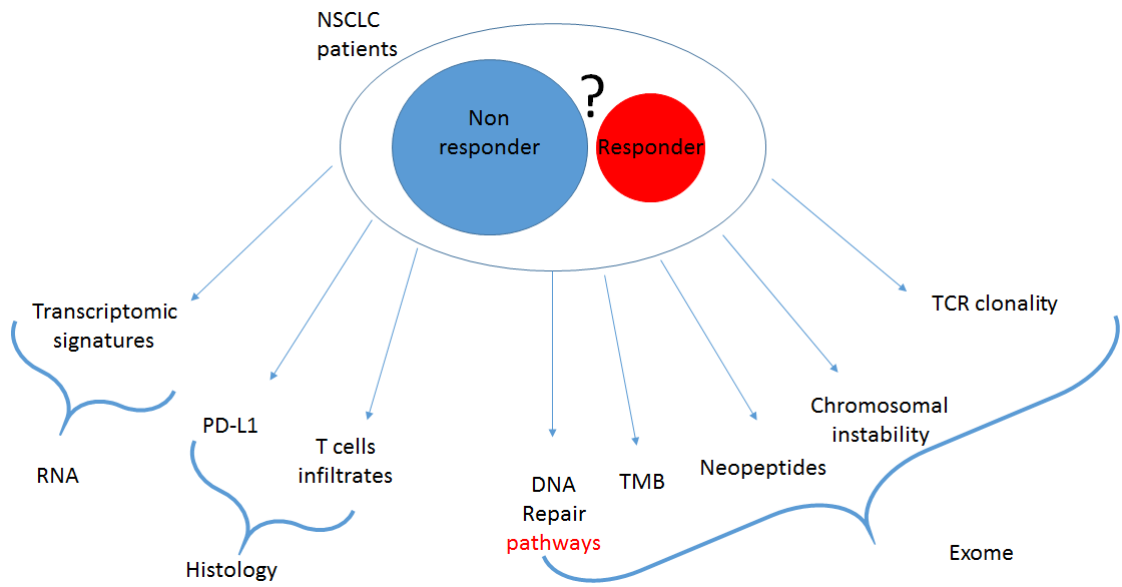
current putative biomarker of response to checkpoint inhibitors

Drugs targeting immune checkpoint inhibitors (CKI) are currently revolutionizing the treatment of lung cancer. Monoclonal antibodies targeting PD-1/PD-L1 demonstrate a spectacular efficacy in different clinical settings. Currently, these drugs can be given as monotherapy in non-small cell lung cancer (NSCLC) in second line or in first line in patients with more than 50% of expression of PD-L1 in cancer cells [1, 2]. These drugs can also be used in association with chemotherapy in either NSCLC or in small cell lung cancer (SCLC) [3, 4]. However, such treatments are not effective in all patients and hyper progression was observed, suggesting that patient selection is required for future developments. Currently the only available biomarker is PD-L1 [5]. Presence of this biomarker is enriched in responder population, but due to its dynamic and heterogeneous expression this biomarker does not allow the exclusion of patients who have no chance to response to CKI. Transcriptomic signature related to IFNγ expression and presence of an immune infiltrate detected by histology are also proposed as putative biomarkers, nevertheless these markers are correlated with PD-L1 expression. In addition to PD-L1, a new biomarker is upcoming, the Tumor Mutational Burden (TMB). This biomarker is defined by the number of all mutations detected in tumoral DNA (synonymous and non-synonymous) per MegaBase of genome examined. TMB could be estimated using Exome or large panel Next Generation Sequencing. High TMB is associated with better response rate, progression-free and overall survival [6]. Exome analysis provides numerous additional data other than TMB, indeed it can be used to determine mutation list. Some particular mutations have an impact on the immune system and could influence the response to immunotherapy. Previous reports suggested that mutations in DNA repair genes, in particular mutations in the homologous repair pathway, are associated with response to CKI [7]. Mutations in mismatch repair genes or in the microsatellite instability signature are also a valuable predictor of response to CKI [8]. In addition, Exome analysis provides genetic information such as the type of mutation signatures, the presence of structural DNA anomalies like aneuploidy, chromosomal instability, amplification/ deletion and presence of neoepitopes generated by mutations. The presence of a high number of mutations in tumors is associated with a higher chance to generate neoepitopes which could be detected by T cells leading to an antitumoral immune response [9]. In addition, it is also possible to estimate the presence of TCR clones in the tumor sample by analysis of CDR3 rearrangement of the TCR alpha or beta genes. It is plausible that such information would improve our ability to predict patient outcome (Figure 1).

It can be expected that a complete analysis of all information found in Exome sequencing would improve outcome prediction in comparison to TMB alone. This question was addressed in the manuscript by Richard *et al* [10]. Somatic and constitutional exome analyses were performed for 77 patients with NSCLC treated with Nivolumab. Tumor-related characteristics such as aneuploidy, tumor copy number alterations clonality, mutational signatures, TMB, mutations in Type I IFN, WNT, AKT, MAPK and DNA repair pathways, and immunological characteristics such as number of intratumoral TCR clones, HLA type and number of neoantigens were studied. Univariate analysis showed that high TMB, a high number of neoantigens, specific mutational signatures, mutations in DNA repair pathway and a low number of TCR clones are associated with a greater outcome. Using the LASSO (least absolute shrinkage and selection operator) method, an approach adapted to the high-dimensional setting, we have estimated that an exome-based model with 9 parameters could discriminate between patients with good or poor outcome without using clinical data. Importantly, this model had an improved ability to predict outcomes when compared to PD-L1, clinical variables or TMB only models. The model was externally validated in a cohort of 34 patients treated with Pembrolizumab [9].

Data presented are the proof of concept that complex analysis of Exome or large NGS panel could outperform TMB for predicting the efficacy of CKI in NSCLC. These new approaches may be the new highways for precision medicine in oncoimmunology.
